# Complications after surgical management of distal lower leg fractures

**DOI:** 10.1186/s13049-016-0333-1

**Published:** 2016-12-09

**Authors:** Mirjam V. Neumann, Peter C. Strohm, Kilian Reising, Joern Zwingmann, Thorsten O. Hammer, Norbert P. Suedkamp

**Affiliations:** 1 Department of Orthopaedic and Trauma Surgery, Albert-Ludwigs-University of Freiburg, Medical School, Hugstetterstrasse 55, 79106 Freiburg im Breisgau, Germany; 2 Department of Orthopaedic and Trauma Surgery, Sozialstiftung, Bamberg, Germany

**Keywords:** Distal tibiofibular fracture, Postoperative complication, Intramedullary nailing, Plate fixation

## Abstract

**Background:**

Osseous healing of distal lower leg fractures can be prolonged and is often associated with wound healing problems because of the marginal soft - tissue and vascular supply in this area. Postoperative complications are frequent, and according to the literature, open reduction and plate fixation is thought to be associated with higher complication rates. The objective of this study was to evaluate the most common postoperative complications following intramedullary nailing or plate osteosynthesis of distal lower leg injuries with a focus on combined tibio-fibular fractures. The outcomes of patients with and without complications associated the two surgical techniques were compared.

**Methods:**

During a 5-year period, all surgically treated distal tibiofibular fractures were retrospectively collected from the clinical database and were evaluated for the presence of postoperative complications which included compartment syndrome, wound infection, delayed union and non-union, synostosis and rotational malalignment. Postoperative complications were reviewed and correlated with patient risk factors.

**Results:**

A total of 199 patients were included in the study, and 75 complications were reported. The majority of complications were associated with closed fracture types treated with intramedullary nailing, delayed union being the most frequent. For open fractures, surgical treatment with plate fixation had a complication rate of 12% compared with 25% after intramedullary nailing.

**Discussion:**

In general, distal lower leg fractures are associated with a high risk of postoperative complications. Distal diaphyseal tibial fractures that have been treated with intramedullary nailing devices have a higher risk of delayed union or non - union.

**Conclusion:**

Plate fixation in distal metaphyseal fractures has a higher risk of problems related to wound healing and postoperative wound infections.

## Background

Lower leg fractures have a reported incidence of up to 184 fractures per 100.000 persons per year [1]. Surgical management and postoperative care can be challenging, primarily because osseous and wound healing of the distal lower leg can be critical. The blood supply of the lower leg runs axially leading to a dysbalance of the intramedullary blood supply to the disadvantage of the distal tibia [2]. The surrounding soft tissue is vulnerable as covering muscles are missing, leading to a reduced osseous healing capacity as well [2].

Though metaphyseal tibial fractures rarely occur with fibular fractures [3], fibular fractures with distal, diaphyseal tibia fractures, that are mainly located in the supratubercular or media diaphyseal regions have an incidence of > 75% [4]. Surgical treatment of distal tibiofibular fractures with intramedullary nailing (IMN) is the preferred method of repair [5], even in very distal fractures [6]. Some studies have even popularized intramedullary nailing in open fractures [5, 7, 8]. With the introduction of the MIPO technique (minimally invasive plate osteosynthesis) and new plate designs that function as internal fixators [9], plate fixation of tibia fractures gained renewed attention [10, 11]. Some authors actually recommend using plate and screw fixation to repair distal tibiofibular fractures [12, 13].

It is well known that postoperative complications are associated with intramedullary nailing or internal plate osteosynthesis, however the focus has been primarily on non-union or wound infections [10, 14, 15]. Reported outcomes following the surgical management of distal lower leg fractures refer frequently to tibial fracture fixation, especially in defined pilon fractures, and the associated complication rates [16, 17]. However, few studies address the treatment of combined distal tibiofibular fractures [4, 18, 19].

The objective of this study was to compare postoperative complications in distal lower leg fractures managed with intramedullary nailing systems with those managed with plate osteosynthesis. We hypothesized that complications occur more frequently in open fracture types and after open reduction and plate fixation. This study focused on the correlation between outcomes and surgical management, patient risk factors and complication rates, as well as open fracture types and their complication rates.

## Methods

In this retrospective study, all distal tibiofibular fractures (AO/AO – ASIF (Association for the Study of Internal Fixation) types 42-A1, A2; 42-B1, B2; 42-C1; 43-A1 - A3, B2, B3, C1 – C3) surgically treated between January 2010 and December 2014, were reviewed. Data were collected from the clinical database.

Surgical selection criteria for the management of distal tibio-fibular fractures followed the department’s internal treatment scheme. For open and closed fractures type Gustilo Anderson II and higher [20], a staged procedure with the initial placement of an external fixator was indicated, while fractures with no soft-tissue damage were managed by immediate definite fracture fixation. All fractures were surgically treated within a minimum of 24 to a maximum of 48 h post injury. The choice of implant was depended on the fracture location and the type of fracture. Complex distal fractures e.g., pilon tibial fractures, were generally fixed with a plate, while tibial shaft fractures that extended up to a location above the epiphyseal plate were managed using unreamed intramedullary nailing devices.

Analysis focused on radiographic results and a review of patient charts. Exclusion criteria were paediatric fractures, patients with multiple injuries and patients with incomplete clinical and-/or radiographic charts. No clinical examination was performed at the time of the data analysis.

Classification of open fracture types followed the Gustilo Anderson definition of soft-tissue damage [20].

Delayed osseous healing was defined as the absence of signs of osseous healing 16 weeks after surgical intervention. A definite point in time for non-union is difficult to define because multiple cofactors are involved in the genesis of non-union. In this study, non-union was defined when there was no osseous consolidation seen 6 months postoperatively. The final outcome and final follow-up were defined as either documentation of completed osseous healing on plain radiographic films or the time of implant removal.

Statistical analyses were performed using Intercooled Stata Version 12 (StataCorp LP, TX, USA), and statistical significance was set at *p* < 0.05.

## Results

In total, 348 patients were treated for distal tibiofibular fractures between January 2010 and December 2014. Moreover, 124 patient charts were excluded because their follow-up was performed in other clinics. Thus, 199 patients charts were retrospectively reviewed for data analysis. Demographic and general outcome data are presented in Table 1.

**Table 1:** Presentation of the general data outcome and the number of chosen fracture management

General data outcome		
gender (n)	female	66
	male	133
age (years)		46 (15–92)
total follow-up (months)		18 (2–102)
time to bone healing (months)	no comorbidities	5.4 (2–9)
	comorbidities	7.25 (3–12)
ORIF (n)	IMN	103
	angular locking plate	58
	LCP + screw	22
	definite Ex. Fix.	5
	other	11

In total, 72 postoperative complications were identified in 67 patients. Complications during follow-up were allocated to early and late complications and differentiated into wound infection, compartment syndrome, delayed osseous healing; non-union, ankle valgus deformity and postoperative synostosis.

Significantly more complications were found in those cases treated with an intramedullary nailing device (*p* < 0.006) (Table 2). In general, osteosynthesis with an IMN was associated with significantly more complications for closed fractures (*p* < 0.0224).

**Table 2:** Distribution of complications after intramedullary nailing and plate fixation of distal tibiofibular fractures

Complication	IMN	Plate Fixation
Wound Infection	2	9
Compartment Syndrome	7	0
Delayed Osseous Healing	19	0
Valgus Deformity	0	5
Synostosis	8	5
Non-Union	8	4
total	44	28

Development of a postoperative compartment syndrome may be due to preoperative soft tissue conditions^†^significance (*p* < 0.05), *p* < 0.006

^†^ Wilcoxon Test

Postoperative complications for all fractures were significant for distal diaphyseal tibial fractures treated with IMN (*p* < 0.001) (Table 3). A significant difference was found in the management of metaphyseal and diaphyseal lower leg fractures between open and closed fracture types (*p* < 0.0001) (Table 3).

**Table 3:** Differentiation of fracture entities and the frequency of soft tissue damage and rate of complications

	Group 1: Distal Metaphysis Fracture of the Lower Leg		Group 2: Distal Diaphysis Fracture of the Lower Leg		*p*-value between groups
	IMN	Plate Fixation	IMN	Plate Fixation	
Soft Tissue Damage					
Closed Type I	6	23	10	4	
Closed Type II	7	13	9	1	
Closed Type III	0	0	2	1	
n	13	36	21	6	*p* < 0.0001^†^
Open Type I	1	6	7	0	
Open Type II	3	11	11	1	
Open Type III					
IIIa	0	0	7	1	
IIIb	2	1	3	1	
IIIc	1	0	1	0	
n	7	18	29	3	*p* < 0.0001^†^
Complications					
Wound Healing	0	5	0	0	
Wound Infection	1	8	1	1	
Compartment Syndrome	1	0	6	0	
Valgus Deformity	0	5	0	0	
Delayed Union	0	0	19	0	
Non-Union	5	3	3	1	
n	7 (35%)	21 (38%)	29 (58%)	2 (20%)	

Significance (*p* < 0.05), *p* = 0.0252^†^, *p* < 0.001^†^, *p* = 0.0251^†^

^†^ Wilcoxon Test

Significantly more postoperative complications were associated with plate fixation for distal metaphyseal fractures (*p* = 0.0252) compared with all fractures (Table 3). In general, there was a significant difference in postoperative complications between distal metaphyseal and distal diaphyseal tibial fractures (*p* = 0.0251) (Table 3). There was no significant difference in postoperative complications between open and closed fractures.

Preliminary fracture reduction and stabilisation by placement of an external fixator was performed in 100 cases, of which 40 were open fractures. There were no significant differences in outcomes between preliminary fracture stabilisation using an external fixator and definite fracture fixation using plate osteosynthesis (*p* = 0.2605) or IMN (*p* = 0.8472), either with open or closed fracture types.

Time to osseous healing was on average 5.4 months (2–9 months) for all patients without significant comorbidities. Patients with comorbidities had a documented prolonged osseous healing time of 7.25 months (3–12 months).

**Fig. 1:**
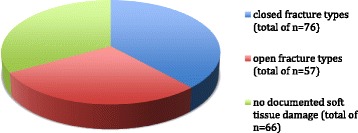
Distribution of soft tissue damage for all reviewed distal tibio-fibular fractures

Three patients died during treatment, each following cardiovascular complications. In two cases, lower leg amputation was necessary after a Gustilo Anderson type III C open fracture. This was necessary in a 56-year-old male patient and in an 88-year-old female patient who were affected with peripheral arterial dysfunction Grade IV 2 months following the surgical repair. Overall, 133 patients sustained fractures with documented soft-tissue damage. A total of 57 open fractures (Gustillo and Anderson classification) were included (Tables 1 and 3, Fig. 1).

Eleven patients were known to be alcohol addicted, 6 patients had a diabetes, and 3 patients showed peripheral arterial dysfunction Grade IV. The number of smokers was not surveyed.

The selective analysis between pilon tibial fractures and metaphyseal fractures showed no significant difference in fracture management (*p* = 0.8755) and gender allocation (*p* = 0.1288). There were no differences in age distribution (*p* = 0.2684), distribution of patient risk factors (*p* = 0.3573) or postoperative complications (*p* = 0.5463) (Table 4).

**Table 4:** Subanalysis of metaphyseal tibia fractures comparing fracture management, demographic factors, patient risk factors and postoperative complications

Fracture Entity	Fracture management			Gender		Age	Patient’s risk factor			Postoperative complication		
*Pilon tibial fracture*	pre-definite fracture fixation with Ext Fix	Plate ORIF	IMN	male	female		Alcohol dependency	Diabetes mellitus	Peripheral arterial disease	Wound infection	Compartment syndrome	Non-union
open type I	0	1	0	1	0	56						1
open type II	4	4	0	2	2	50 (41–57)				1		
open type III	0	0	0	0	0	0						
closed type I	11	10	0	7	4	51 (16–85)	2			3		1
closed type II	3	4	0	2	2	56 (49–54)						
closed type III	0	0	0	0	0	0						
no soft tissue documentation	7	12	0	10	2	41 (23–73)	1					
*n* = 32						Ø 53						
*Metaphyseal distal tibial fracture*												
open type I	2	0	1	1	1	65 (59–70)	1		1	1		
open type II	0	0	0	0	0							
open type III	1	0	1	0	1	56				1	0	1
closed type I	5	6	0	4	4	54 (45–65)	2			1		1
closed type II	6	5	2	1	6	51 (21–72)		2		3	1	1
closed type III	0	0	0	0	0	0						
no soft tissue documentation	3	2	2	4	0	46 (28–56)	4			1		1
*n* = 22						Ø 52						
significance between groups _unpaired *t*-Test_		*p* = 0.8755		*p* = 0.1288		*p* = 0.4132	*p* = 0.3573			*p* = 0.5463		

All of the patients with peripheral arterial dysfunction (*n* = 3) developed postoperative wound infections. Alcohol addicted patients (*n* = 7) had no higher risk of postoperative complications, while 50% of patient’s with diabetes mellitus (*n* = 5) developed postoperative complications.

**Fig. 2:**
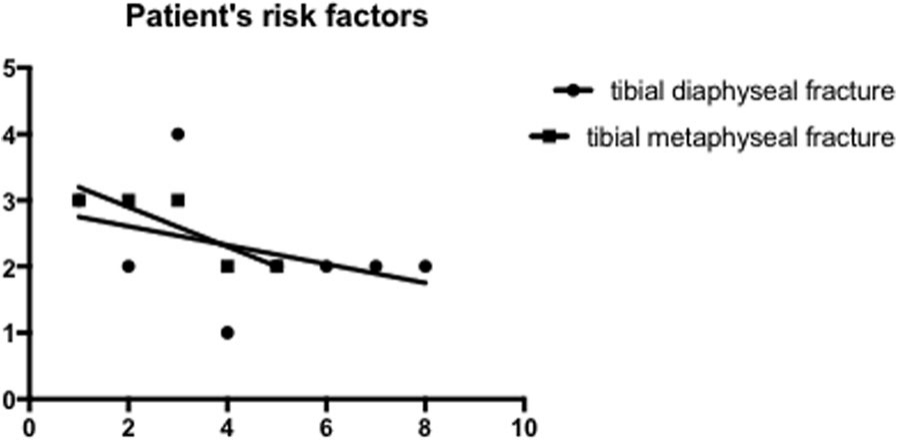
Linear regression analysis for fracture patterns and patient factors in distal lower leg fractures

Regression analysis evaluating population characteristics in relation to fracture patterns did not reveal any significant correlations (*p* = 0.4481) (Fig. 2).

Implant removal was required in 102 patients after a mean of 16 months (range 2–112 months). Two early implant removals were necessary due to a local wound infection, while one was performed in a 16-year-old male patient who was disturbed by the implant. The indications for implant removal were similar between intramedullary nailing (*n* = 53) and plate osteosynthesis (*n* = 49).

## Discussion

This retrospective analysis provides an assessment of different surgical fixation methods for the management of distal tibiofibular fractures, based on a considerable amount of data. Hypothetically, open fractures fixed with plate osteosynthesis are difficult to manage. However, the data revealed that the most popular fixation method of distal tibiofibular fractures using an intramedullary nailing device is associated with the highest risk of complications. Delayed osseous union followed by osseous non-union was most often seen after intramedullary nailing (IMN). The fracture location in the tibial diaphysis is often involved when postoperative complications occur, especially after surgical treatment with intramedullary nailing.

Several studies have retrospectively reviewed complications resulting from the management of distal tibiofibular fractures [14, 15, 19]. However, these studies focused primarily on either intramedullary nailing or open reduction and internal fixation techniques. Intramedullary nailing is most frequently associated with different kinds of postoperative complications. This might be because repairing a fracture with IMN is carried out immediately, while plate fixation more often follows a staged procedure after preliminary fracture stabilization with an external fixator. Hence the wound is managed with greater care. In our retrospective analysis the location of the fracture determined the method of fracture fixation. Distal tibia diaphyseal fractures were frequently repaired with IMN (mean distance ephysis to fracture 7 cm (2.1–10.2 cm)), while plate osteosynthesis was used to repair distal metaphyseal tibial fractures.

Preliminary fracture fixation of distal lower leg fractures with an external fixator is the treatment of choice in the management of open fractures types III A, B and C as well as in type III closed fractures. In our review, initial fracture stabilisation by placement of an external fixator was performed in 45% (*n* = 100) of all included cases. Of these, 52% (*n* = 52) were definitively fixed with plate osteosynthesis and fewer complications were seen in this group (*n* = 16) during the postoperative course compared with IMN (*n* = 22) after initial external fixator stabilisation. Outcome bias in the present study may be due to delayed operative management, which is related to the fact that patients tend to present later to our clinic because they are referred from elsewhere. There was no significant difference between the numbers of open and closed fracture type fixations that were initially managed by placement of an external fixator followed by IMN or with plate fixation. But the overall result highlights the better outcome and fewer postoperative complications following a staged procedure used to manage distal lower leg fractures and plate osteosynthesis.

Postoperative compartment syndromes are more often seen after IMN and are also due to immediate posttraumatic fracture fixation. It is not clear whether fracture non-union develops because additional fixation of combined fibular fractures leads to a fracture fixation that is too rigid. Rouhani et al. could not confirm any additional benefit of fixation of fibular fractures in the distal third combined with distal tibia fractures. However, they noted the importance in fixation of the distal third fibular fractures in maintaining lower leg length and rotational stability [18]. In the present study, delayed osseous healing or non-union was observed in 2 and 6% of all complications following IMN and fibular plate fixation, respectively. Consistent with other studies [21], postoperative synostosis was observed in 15% of all complications. In our review, synostosis occurred mainly after IMN, though the difference was not significant.

In our study, rotational malalignment after the management of distal tibiofibular fractures was reported in 8% of cases of postoperative ankle valgus malalignment in distal metaphyseal tibia fractures. This occurred primarily after open reduction and plate fixation and was potentially due to plate design or plate bending. Varus malalignment was not seen in our series.

Pre-existing risk factors such as diabetes or peripheral arterial dysfunction led to the expected number of postoperative complications, which were mainly problems with wound healing, followed by non-union. Between the two main surgical fixation methods, there was no difference in postoperative complications in patients with pre-existing risk factors. Subanalysis of metaphyseal tibial fractures showed significant differences for gender distribution and fracture management. However, there were no differences in outcomes comparing patient risk factors and postoperative complications.

Limitations to this study include the median range number of retrospectively analysed data and the absence of clinical evaluations of the outcomes. Because the end point of follow-up was defined as osseous fracture consolidation or implant removal, the duration of follow-up is not consistent and is dependent on postoperative complications. The total number of patients (*n* = 22) with pre-existing risk factors is low. This may explain why there was no difference in outcome depending on patient risk factors in relation to fracture types or surgical management. Another weakness of the study is that the retrospective study design that cannot control for selection bias. Study outcome bias might be due to the more frequent preliminary fracture reduction with an external fixator and stepwise facture management in the plating group.

The criteria for selecting surgical treatment could also lead to data bias in the compared groups. The imbalance of distributed numbers in the surgical treatment between intramedullary nailing and plate fixation in distal diaphyseal fractures might have lead to an outcome bias presenting more complications after intramedullary nailing. However, our clinic follows a definite treatment scheme, thereby excluding potential data bias.

Overall, 32% of postoperative complications rate following surgical management of distal tibiofibular fractures is comparable with that reported in other studies [15]. In young patients, these complex, multifragmentary and frequently open fracture types are due to a high velocity impact. In elderly patients a complex distal lower leg fracture generally occurs with minor trauma and is due to poor bone quality. Careful fracture analysis (preoperative CT (Computed Tomography) scan), planning and treatment are mandatory. A staged procedure is preferable. Further development of intramedullary nailing systems and insertion points (retrograde nailing) keep this method of fracture fixation attractive even in the fixation of very distal lower leg fractures [6]. Smaller skin incisions, easier postoperative care and early weight bearing are the main advantages of intramedullary nailing devices. However, modern plate designs and techniques offer reasonable and comparable results after plate fixation of distal lower leg fractures, and can also be performed with a minimally invasive approach.

## Conclusions

Surgical management and postoperative care of distal tibiofibular fractures is challenging, mainly because of the limited vascular supply in this area. Open fractures are generally considered to have a higher risk of postoperative complications. However, our results show that, in general, treatment of distal lower leg fractures with an intramedullary nailing device is associated with a higher number of postoperative complications, mainly delayed osseous healing. Fractures with soft-tissue damage of the distal diaphyseal tibia treated with intramedullary nailing are associated with a higher risk for delayed osseous healing. In contrast, plate fixation in distal metaphyseal tibial fractures and soft tissue damage is often associated with postoperative wound healing problems and wound infections.

Pre-existing risk factors such as diabetes and peripheral arterial dysfunction account for the majority of postoperative complications following different surgical techniques in the management of distal tibiofibular fractures. A staged procedure with preliminary fracture stabilization by placement of an external fixator followed by definite fracture fixation after soft-tissue conditioning is recommended for these often challenging fracture types.

## Abbreviations

AO/ASIF: Association for the Study of Internal Fixation
CT: Computed Tomography
IMN: Intramedullary Nail
LCP: Locking Compression Plate
MIPO: Minimal Invasive Plate Osteosynthesis
